# Clostridioides difficile detection and infection in children: are they just small adults?

**DOI:** 10.1099/jmm.0.001816

**Published:** 2024-03-25

**Authors:** Sam Watkin, Francis Yongblah, James Burton, John C. Hartley, Elaine Cloutman-Green

**Affiliations:** 1 Department of Civil Environmental and Geomatic Engineering, Healthy Infrastructure Research Group, University College London, Chadwick Building, London, UK; 2 Great Ormond Street Hospital NHS Foundation Trust, Camelia Botnar Laboratories, Department of Microbiology, London, UK

**Keywords:** colonisation, *C. difficile*, diagnostics, outbreaks, paediatrics

## Abstract

*Clostridioides difficile* is a well-recognized healthcare-associated pathogen, with its significance widely recognized in adult populations. Despite this, there is limited data on the significance of detection within paediatric populations, both for individual patient management and wider transmission risk-based considerations. High rates of colonization are understood to occur in infants, with increasing levels up to 11 months, and colonization rates similar to adults by 8 years old. Sources of *C. difficile* are ubiquitous, with detection in companion animals and food sources, as well as within the clinical and wider environment. Due to the close interactions that occur between children and the environment, it is understandable that increasing recognition is afforded to the community acquisition of *C. difficile* in children. Other risk factors for the detection of *C. difficile* in children are similar to those observed in adults, including prior hospitalization and underlying conditions affecting gut health and motility. Recent studies have shown rising awareness of the role of asymptomatic carriage of *C. difficile* in healthcare transmission. Prior to this, paediatric patient populations were less likely to be screened due to uncertainty regarding the significance of detection; however, this increased awareness has led to a review of possible carriage testing pathways. Despite this increased attention, *C. difficile* infection remains poorly defined in paediatric populations, with limited dedicated paediatric data sets making comparison challenging. This is further complicated by the fact that infection in children frequently self resolves without additional therapies. Due to this, *C. difficile* remains a management challenge in paediatric settings.

## Introduction

*Clostridioides difficile* is a spore-forming anaerobic Gram-positive rod that is found in both animals and the environment. The human pathogenic potential of *C. difficile* was first described in the 1970s and since the 2000s has been recognized within Western nations as a leading cause of healthcare-associated infection in adults. There is a paucity of data in low-income countries to fully understand global prevalence and impact [1,2]. Recent prevalence in western healthcare systems has been predominantly driven by hypervirulent ribotypes, for example, ribotype 027 (strain BI/NAP1/027) [3]. Several other strains are known to be associated with *C. difficile* infection (CDI) however, for example ribotype 023 [4].

In addition to detection in humans, *C. difficile* has been identified in farm, wild and companion animals, where asymptomatic carriage is estimated at between 11% and 40 % [5]. *C. difficile* can also be recovered from the human food chain, with detection in 20%–63 % of USA/Canadian meat products and 0%–6.3% recovery from studies of European meat products [5]. *C. difficile* has further been isolated from numerous environmental reservoirs, including water associated with farms (36 %), soil (21 %) and healthcare surfaces (20 %), potentially due to the environmental stability and decontamination resistance of *C. difficile* spores [5,6]. The hypervirulent strain BI/NAP1/027 has been recovered from the domestic environment from kitchen and toilet sites [7]. This widespread distribution indicates a One Health approach may be required to improve understanding of transmission routes outside of healthcare settings.

In children *C. difficile* is increasingly recognized as a significant aetiological agent, as well as being an age-dependent colonizer of the paediatric gut microbiome with similar risk factors for progression to infection as those seen in adult populations. This progression is linked to inflammation and damage of the colonic mucosa due to the release of proteinaceous toxins produced by toxigenic *C. difficile* strains [8]. Historically, it was believed that the predominant route of spread in healthcare was linked to symptomatic patients; however, new typing methods with higher levels of discrimination now suggest that asymptomatic carriers may have a significant role in transmission. This is an important consideration within paediatric settings as risk of colonization with *C. difficile* may be linked to the environment in which a child develops [5,9].

## Epidemiology

### Differences between *C. difficile* colonization and infection

*C. difficile* is understood to colonize both the adult and the infant gut at different frequencies. The definitions linked to the term * C. difficile* colonization vary across the literature. A recent article on *C. difficile* colonization utilized ‘the detection of the organism in the absence of *C. difficile* infection symptoms’ as a definition of colonization [5]. Other definitions have included the requirement for no symptoms of CDI within the 12 weeks pre-or post-sampling, or the need for samples to be taken at multiple time points with no symptoms of CDI at the time of sampling [5,10]. Issues with this definition of colonization exist as the number and quality of samples collected in published literature can be highly variable.

One reason for variable sampling strategies and definitions of colonization is due to the intended aim of the sampling, for example, to determine if colonization is transient or persistent. Surveillance can also be conducted to assess potential onward transmission risk or for patient management. In order to better determine the true burden of *C. difficile* infection and colonization in paediatric populations, these two states must be clearly defined (Table 1). Distinguishing between the two states is important for detection and management of *C. difficile* in paediatric populations, due to the difficulty of distinguishing *C. difficile* infection from coincidental colonization detection, where symptoms are due to a separate clinical issue.

**Table 1.** Proposed definitions of *C. difficile* colonization and infection

Clinical state	Definition
*C. difficile* colonization	A new episode of multiplication of *C. difficile* in the gut, which does not lead to harm
*C. difficile* infection	A new episode of multiplication of *C. difficile* in the gut that (i) was not previously present, leading to harm (referred to as disease) or (ii) was previously present and not causing harm (colonization), but now is leading to harm

### *C. difficile* colonization in children

Reported rates of *C. difficile* colonization vary widely with rates reported from 4%–71 % in the under 2's, with one study reporting 90 % of infants and newborns as having detectable *C. difficile* in their stool [5,11]. This variation is potentially due to definitions used but may also be due to the subject populations. Average colonization rates of infants <1 month old have been identified as being 37 % in one study [12]. Other studies suggest colonization is age dependent with a peak of colonization detection around the age of 11 months, with 35%–40 % of the under 1's carrying either toxigenic or non-toxigenic *C. difficile*, reducing to levels of around 3% seen in those age 8 and above, similar to that seen in adults [13,14, 15]. There are however reported lower rates, with one study detecting 48/324 (14.8 %) neonates colonized with *C. difficile*, of which only 53.3 % of recovered strains were detected as toxigenic [15]. Additionally, the lack of longitudinal data, both for adults and children, makes it challenging to understand the stability of any detected colonization within cohorts [5].

In a study by Stoesser *et al.* 2017, factors associated with community colonization included exposure to pets and childcare workers/institutions, although pet colonization could be a marker for wider household colonisation. Breastfed children were less likely to be colonized, although it was unclear whether this was linked to reduced risk of ingestion, or immunological effects on the developing microbiome [13]. Other factors that increased colonization risk included antibiotic consumption and Caesarean delivery [8,13].

### *C. difficile* infection

The term CDI has been used widely and variably in publications linked to detection of *C. difficile* in symptomatic patients. This is not a standardized term however, which can lead to uncertainty due to varied detection methods and symptom profiles. In order to confirm CDI, a combination of laboratory detection, symptom review and risk factor assessment must be undertaken. Clinical presentations in children are similar to those seen in adults, from self-limiting diarrhoea to more rarely severe complications, such as pseudomembraneous colitis and toxic megacolon [8,16]. Due to the frequent detection of confounders, such as non-infectious diarrhoea, differentiating children who require management linked to CDI from those colonized with toxigenic strains remains a challenge however [8,17].

CDI studies within paediatric populations are limited but have primarily demonstrated community-onset, with rates of community-onset CDI being shown to be 17.9 per 100000 in a 2011 USA paediatric cohort, with evidence suggesting increases in rates [18,19]. A recent multi-centre European study in contrast reported a CDI incidence in children of 1.5 % in the 2–18 age category [20]. There is however variability in data collection, testing protocols and study setting between centres and countries, especially in the under 2's, which must be considered when comparing rates [2].

As in adult populations, antimicrobial exposure is understood to be a major risk factor for paediatric CDI. This is due to the impact antimicrobials have on the gut microbiome, allowing *C. difficile* to establish and persist in the gut, or overgrow if already established. Both quinolone and non-quinolone antibiotics have been identified as risk factors for paediatric CDI, with multivariate odds ratio (95 % confidence intervals) of 17.04 (5.86–49.54) and 2.23 (1.18–4.20), respectively [21]. Not all paediatric CDI cases are associated with antibiotic exposure however – one study of 200 children with *C. difficile* associated diarrhoea identified 74.5 % as having had antimicrobial exposure in the previous 2 months [22]. Other factors identified as increasing risk of CDI, excluding advanced age, are similar between adults and paediatrics (Table 2). Some of these may be markers for other correlated risks however, such as underlying immunosuppression or antibiotic exposure.

**Table 2.** Identified risk factors for paediatric CDI. Known risk factors for CDI are listed, with the mechanisms driving increased risk given if known

Risk factor	Potential mechanism of increased risk	Reference
Hirschsprung disease (or other severe gut motility disorders)	Unclear mechanisms	[86]
Gastric acid suppression therapy	Reduction in stomach acid levels removes a protective barrier against *C. difficile* progressing to the intestine; potential alterations to the gut microbiome	[87,88]
Calcineurin inhibitor immunosuppressants (in solid organ transplants)	Unclear mechanism	[89]
Inflammatory bowel disease	Potential microbiome alterations, chronic colonic mucosa inflammation and associated antimicrobial therapy	[90,91]
Tracheostomy dependence	Potential association with increased length of stay and antimicrobial usage	[92]
Presence of malignancy	Increased risk due to increased likelihood of antimicrobial and cytotoxic therapy	[90,92, 93]
Length of hospital stay	Increased likelihood of antimicrobial therapy, increased environmental exposure	[94]

### Molecular epidemiology

*C. difficile* has a global distribution, with different ribotypes and sequence types being dominant in different regions [2]. Despite this, the majority of data on *C. difficile* in paediatric populations arises from western settings. In general, there is a lack of published epidemiology of CDI within low-income countries, this may be linked to restricted access to testing and typing resources, especially within the African continent [2]. There is also relatively little known about the distribution of paediatric CDI in Latin and South American countries, outside of a few reports linked to prevalence of ribotype 027 in Costa Rica [2,23].

There are a number of different molecular typing techniques utilized to undertake *C. difficile* surveillance, with the main approaches being split into fragment-based or sequence-based approaches [9]. Commonly utilized fragment-based approaches differ between North America and Europe, with North America predominately utilizing pulse field gel electrophoresis (PFGE) or restriction endonuclease analysis (REA) for typing, whereas Europe maintains a reliance on PCR ribotyping as a primary method [2,9]. PFGE can discriminate some strains better than PCR ribotyping, however ribotyping has the advantage that a harmonized standard method has been validated across North America and Europe, permitting high-quality transferable results. Although PFGE was adopted as the primary typing scheme utilized by the CDCs within Canada and the USA, both have now switched predominantly to sequencing-based approaches utilizing multi-locus sequence typing (MLST) or whole genome sequencing (WGS) [9,24].

A summary of the molecular characterization of *C. difficile* strains is shown in Table 3. As homology between sequence types and ribotypes is not exact, with some ribotypes aligning to multiple sequence types and vice versa, it has therefore been suggested that where ribotyping is incapable of detecting strain differences, MLST can be utilized [9]. Caution should also be applied for some sequence types when determining recurrence or treatment failure, as some sequence types may not adequately distinguish between ribotypes leading to clinical interpretation challenges and over-calling relapse rates (Table 3) [9].

**Table 3.** Molecular characterization of *C. difficile* epidemiology. Hypervirulent ribotypes are in bold italic. Ribotypes which feature under multiple sequence types are in bold. Table adapted from [9] with additions from [95]

Clade	Clade features	Sequence type	Ribotype
Multi locus sequence typing – sequence analysis of seven housekeeping genes			PCR Ribotyping – analysis of the 16S-23S intergenic spacer region
1	The most heterogenous clade containing the greatest number of sequence types and ribotypes	2	005, **020/014**, 015, 069, 076, 095, 220
		3	001, 009, 055, 072, 077, 115, 262, 305
		10	015
		17	018, 052
		33	**014/020**, 064, 216, 369
		34	056
		35	002, 046, 220
		42	106, 118, 174
		44	015, 062
		45	013, 017
		54	012, **014/020**
2	Clade has low recombination rates and contains hypervirulent ribotype 027	1	002, 003, 016, ***027***, 036, 176
		41	46, 106, 156, 164, 194, 208, 209, 244, 321
3	Contains clade-specific Tn6218 insertion mutation in the pathogenicity locus and presence of pathogenic ribotype 023	5	023, 063, 069, 122, 438
		22	023
4	Clade has a high multidrug resistance prevalence	37	017, 047
		81	PKI-017, A
5	Clade has low recombination rates and contains hypervirulent ribotype 078	11	033, 045, 066, ***078***, 126, 127, 193, 237, 280, 281
I	Cryptic clades characterized by atypical variants of toxin genes (*tcdA*, *tcdC*, *cdtA*, *cdtB*)	181, 206	Not studied
I and II		946, 947, 948	151
II		200	Not studied
III		369	Not studied

As more centres move to utilization of WGS or whole genome MLST, there has been increased awareness that some of the assumptions linked to sources and transmission of *C. difficile*, especially within the nosocomial setting, may be incorrect. Recently some studies have noted that, unlike previously believed, a large proportion of cases cannot be linked via transmission chains to symptomatic cases, and re-assessment of potential sources of *C. difficile* is required, including how asymptomatic carriage may contribute to disease epidemiology [5,10].

## Pathogenesis

Initial ingestion of *C. difficile* occurs through the faecal-oral route, with exposure originating from animal, environmental and healthcare sources. Once ingested, *C. difficile* spores are able to withstand the acidic conditions in the stomach, and progress through the gastrointestinal system [24]. In order to establish colonization and infection, these spores must germinate to metabolically active vegetative cells. The ability of *C. difficile* spores to germinate is determined by the complex balance of bile salts present in the intestine. As summarized by Lawler *et al.* [25], the primary bile salt taurocholate is a potent spore germinant, promoting *C. difficile* spore germination. Under normal conditions in the gut with an unaltered microbiome, primary bile salts are converted to germination-inhibitory secondary bile salts, such as lithocholate, reducing the ability of *C. difficile* to colonize the gut. This conversion is facilitated by the microbial communities present in the small intestine. Alterations in the intestinal microbial communities responsible for bile salt processing can therefore reduce this conversion, causing taurocholate accumulation, driving *C. difficile* spore germination [25]. Disruptions in paediatric gut microbiota have been associated with CDI, with an absence of *Ruminococcaceae*, *Lachnospiraceae*, *Bacteroides* and *Porphyromonadaceae* observed in CDI patients. Elevated *Proteobacteria* abundance has also been observed in recurrent CDI (rCDI) patients compared to healthy cohorts [26]. The ability of normal gut microbes to provide protection against *C. difficile* colonization and infection has been further shown through the identification of six potential probiotic *Enterococcus faecalis* isolates. These strains, isolated from the faeces of breastfed infants, both inhibited *C. difficile* spore germination and reduced the impacts of *C. difficile* toxins on HT-29 cells [27].

Once *C. difficile* spores have germinated to vegetative cells, CDI occurs primarily through the production of two toxins, clostridial toxin A (TcdA) and clostridial toxin B (TcdB), both located on the PaLoc locus [24]. These toxins enter colonic epithelial cells via clathrin-mediated endocytosis and interact with the GTPases Rho, Rac and Cdc42, inducing actin condensation, cell rounding and death [28,29]. A minority of *C. difficile* strains located in the cryptic clades produce binary toxin (*C. difficile* transferase – CDT) through the genes *cdtA* and *cdtB* on the CDT locus [9,30]. This toxin further disrupts epithelial cell cytoskeletal structures, increasing the permeability of the epithelial cell wall, leading to extensive fluid loss [28]. The primary toxin TcdA has been described as having strong proinflammatory effects, while single point mutations in the TcdB cysteine protease domain and the cysteine autocleavage site (TcdB-C698S and TcdB-L543A, respectively) increase the proinflammatory effects of TcdB [31]. These effects drive the release of the proinflammatory cytokines interleukin-1β, tumour necrosis factor alpha and interleukin-8, causing neutrophil recruitment and the formation of pseudomembranes on the colonic epithelium [28]. Neutrophil recruitment has been further shown to occur in CDI patients through activation of mitogen-activated protein kinase-activated protein kinase 2, as well as p38 kinase [32]. Some strains of *C. difficile* exhibit increased virulence compared to other toxigenic strains, termed hypervirulence. For example, the strain NAP1/B1/027 possesses a frameshift deletion in the TcdC gene at point 117 – the gene responsible for regulating expression of the TcdA and TcdB toxins – resulting in greatly increased expression of these toxins [3]. Historically, CDI has been discounted in under-2-years-old patient cohorts as a cause of diarrhoeal disease due to a lack of toxin receptors in the neonatal gut. The quality of evidence to support this hypothesis has been challenged however, highlighting the relatively small evidence base informing this belief. This highlights that further research is required into the presence of clostridial toxin receptors in the neonatal and infant gut [33].

As vegetative *C. difficile* cells progress through the large intestine, they are increasingly exposed to environmental stressors, such as nutrient depletion, quorum sensing and the immune response of the host, initiating sporulation [24]. The spores produced are known to be extensively resistant to environmental conditions such as oxygen presence and heat, as well as to chemical disinfectants. This resistance is due to the physical structure of the spore, consisting of a peptidoglycan cell wall, a peptidoglycan cortex and a protein spore coat and exosporium [25]. These spores are excreted into the environment, allowing the cycle of infection and colonization to continue, particularly in the presence of unidentified carriage [24]. Due to the innate resistance of *C. difficile* spores to disinfection, appropriate management of the clinical environment is necessary to prevent onward transmission by supporting load reduction.

## Clinical presentation

*C. difficile* infection is primarily a disease arising from the colon, which is reflected in presentations linked to CDI. As with adult disease, there is a wide range of clinical manifestations in the paediatric setting, from asymptomatic infection (colonization), through self-limiting diarrhoea to severe complications, such as pseudomembraneous colitis and toxic megacolon. However, unlike in adult CDI there is frequently much less severe disease identified in paediatrics. Age is a known risk factor for progression to disease, with conflicting evidence on which age groups exhibit the most severe disease [8,16]. The most frequent clinical presentation has been identified as diarrhoea (typically a short episode of loose stool with a distinctive odour). This may be accompanied by fever, abdominal pain and other non-specific symptoms and signs [8,34]. Severe disease can occur at any age however, with fatal outcomes, although mortality rates have been shown to be 1.0 % in a paediatric cohort study, compared to 24 and 15.3 % in separate adult cohorts [22]. Identifying clinical presentations that indicate infection requiring treatment while avoiding unnecessary interventions is therefore of great importance to support favourable patient outcomes.

Many studies have attempted to define epidemiological, clinical or laboratory parameters which aid in the differentiation of CDI from carriage and from the myriad of other causes of diarrhoea in children. As with adult disease, paediatric patients with underlying conditions, antimicrobial usage, solid organ transplant, haematological/oncological disease and past healthcare contact can present with symptoms of CDI. This combination of risk factor and symptomology is not confirmatory however, and other diagnostic information should be taken into consideration when reviewing the need for treatment.

The specific diagnostic accuracy of symptoms and signs of CDI (in adults and children) have been usefully summarized in a recent meta-analysis by Manzoor *et al.* [34]. Here, the authors included all reports that discuss symptomology of CDI, supported with a reference test to confirm CDI diagnosis. They list and assess the diagnostic accuracy of important commonly reported symptoms associated with CDI (Table 4) [34]. Significantly, while diarrhoea is noted as the most common symptom of CDI, it is not present in all cases and is not specific for *C. difficile*. The authors conclude that there is limited utility of clinical examination alone in detecting CDI. Instead, accurate diagnosis requires clinical assessments to consider symptomology, risk factors and patient cohort as well as the results from microbiological testing in suspected cases [34]. It is important to consider however that due to poor standardization of the term ‘CDI’, the articles included in this meta-analysis may cover symptomatic disease where *C. difficile* is detected but not the true cause of the symptoms.

**Table 4.** Diagnostic accuracy of clinical examinations from studies eligible for meta-analysis. MEDLINE, EMBASE, CINAHL and Cochrane databases were searched to determine the diagnostic accuracies for CDI of different techniques. Table from [34]

Symptom	No. of studies	Total patients (CDI cases)	Sensitivity	Specificity	Positive likelihood ratio	Negative likelihood ratio
Diarrhoea	5	17 143 (1902)	0.48 (0.23–0.73)	0.62 (0.47–0.75)	1.38 (0.64–2.98)	1.15 (0.76–1.73)
Watery diarrhoea	3	1783 (378)	0.72 (0.60–0.82)	0.42 (0.33–0.51)	1.25 (1.02–1.53)	0.66 (0.42–1.01)
Bloody diarrhoea	7	3277 (701)	0.09 (0.4–0.17)	0.92 (0.88–0.95)	1.35 (0.98–1.85)	1.32 (1.02–1.72)
Mucus in stool	2	1310 (274)	0.43 (0.00–1.00)	0.61 (0.01–1.00)	1.29 (0.03–53.82)	1.18 (0.36–3.83)
Abdominal pain	12	10 978 (2454)	0.32 (0.19–0.49)	0.65 (0.51–0.77)	0.91 (0.70–1.19)	1.05 (0.92–1.19)
Abdominal distention	3	544 (285)	0.1 (0.01–0.46)	0.94 (0.83–0.98)	1.8 (0.18–17.65)	1.71 (0.22–13.23)
Ileus	3	464 (169)	0.1 (0.04–0.25)	0.93 (0.84–0.97)	1.57 (0.76–3.26)	1.51 (0.8–2.85)
Nausea / vomiting	5	1802 (255)	0.15 (0.02–0.57)	0.85 (0.36–0.98)	0.95 (0.47–1.92)	0.96 (0.64–1.43)
Fever	13	20 151 (2443)	0.23 (0.12–0.39)	0.82 (0.69–0.91)	1.28 (0.98–1.66)	0.94 (0.87–1.02)
Hypotension	1	74 (50)	0.34 (0.21–0.49)	0.79 (0.58–0.93)	1.96 (0.62–6.16)	1.63 (0.68–3.90)

Although the clinical suspicion of CDI is important in prompting the assessment of paediatric patients, the onward risk of transmission also needs to be considered from both symptomatic and colonized children. A study by Kohler *et al.* [35] identified no significant difference in *C. difficile* spore load in the stool of children in their defined asymptomatically colonized and infected groups [35]. This highlights the importance of identifying patients asymptomatically colonized with *C. difficile* to support infection control practice as well as identifying patients with active infection.

## Diagnosis

The importance of accurate, rapid and effective detection techniques and diagnostic algorithms are well documented and accepted in diagnosing *C. difficile* in the adult population. Debate exists surrounding effective diagnosis of CDI versus colonization in paediatric populations however [28]. Clinical practice guidance for CDI for children in the USA discourages routine CDI testing for the under 2's presenting with diarrhoea unless other causes have been excluded for those aged between 1 and 2 years. These recommendations are based on high reported *C. difficile* carriage rates in neonates and those under 2 [36]. Within guidance in England, it is mandatory to report CDI requiring treatment to national health authorities if the patient is over the age of two. This requirement may therefore drive development and implementation of diagnostic workflows [37]. In general, accurate diagnosis of CDI must include a review of the symptom profile of the patient, including any risk factors, alongside the result from *C. difficile* detection tests alongside other available diagnostic data, such as histology. Organism-specific detection for *C. difficile* in clinical samples can be performed utilizing a variety of methods, with enzyme immunoassays and PCR-based assays frequently being incorporated into diagnostic workflows. Toxin-detecting culture assays have historically been considered the gold standard for toxigenic *C. difficile* detection, however the long test duration required does not fit into standard diagnostic workflows [8,32]. The structure of these workflows may be impacted by national guidance, with guidance in England mandating a two-step detection algorithm with result availability within 48 h [37]. Guidelines in other countries vary, with USA guidance permitting one-step testing algorithms [2,36].

### Detection assays

In order to detect *C. difficile* in stool, enzyme immunoassays (EIA) are often used. These assays look to detect specific markers of *C. difficile* through linkage to antibodies which in turn confer a positive result for the specific analyte. EIA tests for glutamate dehydrogenase (GHD) are used to detect the presence of *C. difficile* in stool. In order to determine toxin competence and production, GDH EIAs are often paired with toxin-detecting EIAs. This allows for the possible detection of *C. difficile* and the presence of *C. difficile* specific toxins, increasing the specificity of the test to currently toxin-producing *C. difficile* [38]. However, this assay will not identify toxin-competent, non-producing *C. difficile*.

An alternative approach to EIAs for *C. difficile* detection is the use of molecular-based assays, such as PCR. PCR assays commonly targeting the *tcdA* and *tcdB* genes have been utilized to detect the presence of toxin-competent *C. difficile* from stool samples [39]. As such methods rely on the amplification of specific DNA targets, they often have improved sensitivity compared to EIA tests. They however do not determine active toxin production or viability and, as such, are not appropriate in isolation for differentiating *C. difficile* infection from colonization [40]. It has also been suggested that the improved sensitivity of one-step molecular assay workflows may contribute to overdiagnosis of CDI in paediatric patients, subsequently leading to unnecessary antibiotic use [41]. Conversely, the use of stool toxin EIAs alone in paediatrics has shown poor performance when compared to direct detection by molecular techniques followed by EIA and culture testing [39]. In order to tackle this, *C. difficile* detection is mandated in England to encompass a two-stage process, whereby EIAs are used to detect the presence of GDH and free toxin, in accompaniment with PCR or toxigenic culture [2,37]. This dual approach to *C. difficile* detection has been shown to have improved diagnostic accuracy in a tertiary care cohort, aiding in the detection of both toxigenic and non-toxigenic *C. difficile* accuracy [42].

**Fig. 1.**
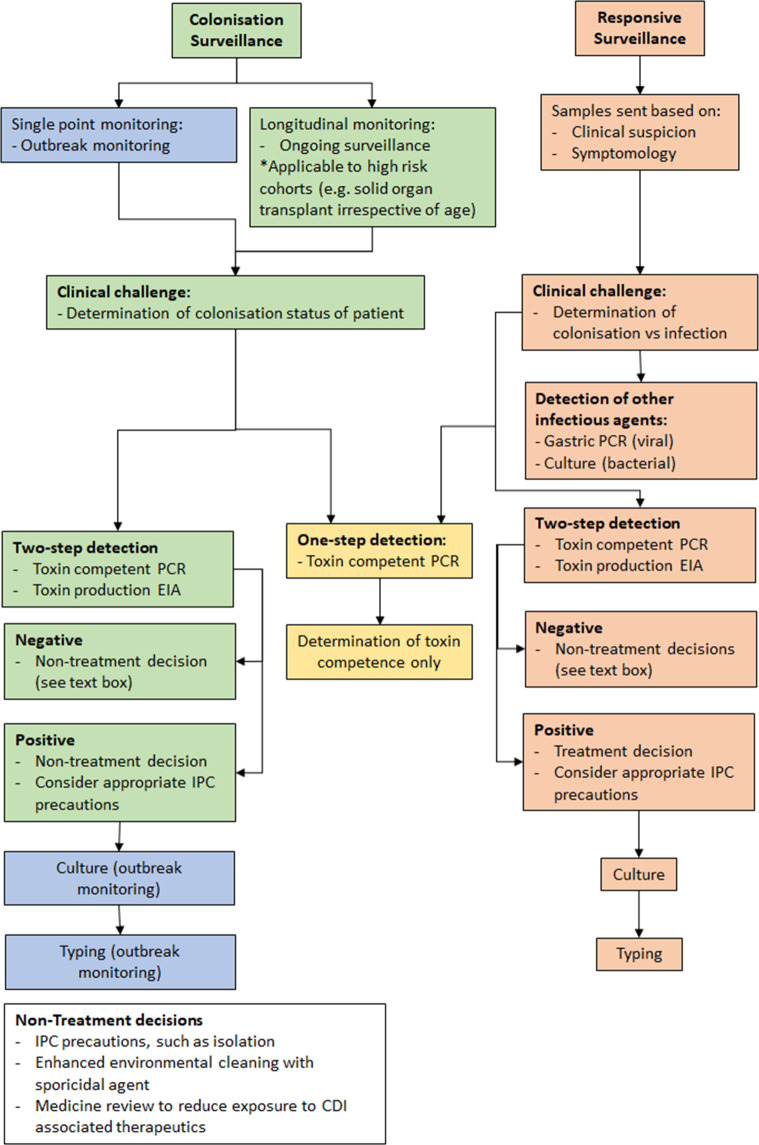
An algorithmic approach to laboratory testing for the presence of *C. difficile* to address different clinical challenges. Potential laboratory investigation pathways for establishing toxin-competent and toxin-producing *C. difficile* from clinical samples. Sample collection is shown to arise from either colonization or responsive surveillance.

The initial decision to test for *C. difficile* should be based on clinical suspicion, based on the patient’s symptomology, clinical history and risk factors, or the clinical risk to others in high-risk settings when aiming to identify colonization. Due to high levels of co-detection of *C. difficile* and other gastrointestinal pathogens, it has been suggested that *C. difficile* testing in ≥2 years old should be combined with testing for other gastrointestinal pathogens to support treatment and reporting pathways [17]. In addition to testing for clinical management, *C. difficile* testing can be performed in patients within at-risk groups in order to identify asymptomatic carriage. This can inform local IPC practice with the aim to limit onward transmission. An algorithmic approach to laboratory *C. difficile* detection testing is described in Fig. 1. This algorithm does not differentiate the decision to test based on patient age (e.g., over or under 2 years old), instead categorizing the reason to test based on determining colonization status or investigating potential CDI. Importantly, diagnosis of CDI should not rely on the outcome of a *C. difficile* detection assay, rather results of detection assays should be interpreted alongside clinical symptoms and existing risk factors.

## Management

As CDI is less frequently seen in paediatric patients compared to adult cohorts, treatment for paediatric CDI is largely based on adult treatment recommendations [17]. As the majority of paediatric CDI cases are self-resolving, treatment is frequently not justified [17,43]. Initial treatment of CDI includes the cessation or review – where possible – of therapies which may exacerbate CDI, such as broad-spectrum antibiotics and proton pump inhibitors [8,44]. Supportive care to manage symptoms is critical in managing paediatric CDI, for example, rehydration therapy [8]. The decision to treat should be based on clinical assessment in combination with the detection of toxin/toxigenic *C. difficile* and an assessment of associated risk factors. While the risk of severe disease in paediatric patients may be less, severe disease can still occur and specific treatment may be required in a small number of cases [19].

### Antimicrobial therapy

Antimicrobial therapy is one aspect of CDI treatment, with several antimicrobial agents available. Current treatment guidelines from the National Institute of Health and Care Excellence (NICE) state vancomycin should be initially used to treat CDI, with fidaxomicin as a second-line therapy and an increased dosage of vancomycin with/without metronidazole as a third-line treatment option [45]. Guidance from the Infectious Diseases Society of America (IDSA) and Society for Healthcare Epidemiology of America (SHEA) currently recommend fidaxomicin be used as a first-line therapy, with vancomycin also viable [46].

A review from Borali and De Giacomo [8] showed oral metronidazole is often used as a frontline therapeutic agent for CDI treatment in paediatric patients. This review highlights the limited treatment options for rCDI in paediatric cohorts, with further courses of metronidazole or vancomycin highlighted as potential options [8]. This is further supported by a survey of American physicians, which found all respondents used oral metronidazole for initial management of mild CDI in immunocompetent patients. Physicians prescribed oral metronidazole less frequently to patients with pre-existing comorbidities however, ranging between 41 and 79% depending on comorbidity. Only 43 % of respondents were found to support *C. difficile*-targeted antimicrobial therapy for *C. difficile* positive infants with recurrent antibiotic exposure and diarrhoea [47]. *C. difficile* resistance to metronidazole has been rarely identified, with one study determining 0.7 % (*n*=1) isolates from colonized neonates as resistant [15]. These studies importantly originate before the publication of updated guidelines from NICE, IDSA and SHEA, with previous IDSA guidance indicating metronidazole to be used as the first-line therapeutic agent in paediatric CDI [48]. Studies supporting this shift away from metronidazole as a first-line therapy for non-severe CDI in paediatric populations have identified earlier resolution of symptoms with oral vancomycin therapy [49]. Both oral vancomycin and oral metronidazole however have been associated with high rates of treatment failure [8,50].

Fidaxomicin is a macrolide antibiotic which has shown efficacy for *C. difficile* treatment in adults and has recently been approved for use in paediatric patients in America, with paediatric dosing guidelines available in the British National Formulary for Children [51]. Fidaxomicin has been shown to have high clinical response rates in paediatric CDI patients. Fidaxomicin has been shown to be well tolerated in paediatric patients, with limited adverse events [52,53]. A phase 3 randomized trial showed higher confirmed clinical response rates in fidaxomicin-treated groups compared to vancomycin treated patients (77.6 % vs 70.5 %), with a statistically significant higher global cure rate in patients treated with fidaxomicin (68.4 % vs 50.0 %) [54]. The cohort investigated in this study predominantly represented adolescent age ranges however, with relatively few (30/148) patients being under 2 years old. The antimicrobial rifaximin has shown similar efficacy to metronidazole for management of paediatric CDI, identifying it as a therapeutic option instead of metronidazole in inflammatory bowel disease patients [55]. Other novel therapeutics for paediatric CDI are under investigation, for example, the TcdB-neutralizing monoclonal antibody bezlotoxumab has recently been assessed for usage in paediatric cohorts [56]. Due to lack of a clear CDI definition in this cohort, it is hard to establish the levels of CDI present in under-2-year-old patient populations, meaning conducting interventional trials is challenging [57].

### Faecal microbial transplantation

The utility of faecal microbial transplantation (FMT) for rCDI management in paediatric populations has been investigated, with one prospective observational pilot study reporting cessation of rCDI symptoms for a minimum of 3 months post-FMT in 12 paediatric patients. Few adverse effects were reported here, with reported abdominal pain attributed to underlying conditions. Significant differences with the pre- and post-FMT gut microbiome were identified, with relative decreases in *Proteobacteria* and increases in

*Bacteroides* post-FMT [58]. A review and metanalysis of the utility of FMT for the treatment of paediatric CDI investigated the findings from 14 studies and concluded that FMT can be both safe and effective for rCDI patients. The authors identify a pooled success rate of 86%, with pooled rates of serious adverse events and adverse events at 2.0% and 15%, respectively. No deaths were attributed to FMT. The authors however discuss how little regulation and standardization exists regarding FMT, further highlighting the lack of randomized controlled clinical trials investigating its utility [59].

### Probiotics

The use of probiotics in the management of *C. difficile* associated antibiotic-associated diarrhoea in paediatric populations has been explored through several studies, with some recommendations on usage published. A report by the European Society for Paediatric Gastroenterology, Hepatology and Nutrition Working Group for Probiotics suggested that *Saccharomyces boulardii* be used if probiotics are considered for preventing *C. difficile*-associated Antibiotic associated diarrhoea (AAD) in paediatric populations [60]. A review published to the Cochrane Library concluded the use of probiotics (S. *boulardii* or *Lactobacillus acidophilus* in combination with *L. casei*) had a protective effect against *C. difficile* associated diarrhoea [61]. Such therapy should undergo appropriate clinical risk assessment however, as probiotic treatment has rarely been associated with fungaemia in immunocompromised patients [62].

## Transmission and outbreaks

While large monoclonal outbreaks of *C. difficile* are seen in adult patient populations, such outbreaks are rarely documented in paediatric cohorts. As surveillance for *C. difficile* colonization and CDI in the under 2's may not be routinely performed, true incidences of paediatric *C. difficile* outbreaks may however be under-reported. Despite relatively little available information, carriage of *C. difficile* has been implicated in onward transmission leading to active disease [63]. A review by Crobach *et al.* [5] showed how multiple studies reported low percentage of strain homogeny in CDI patient cohorts, suggesting a possible role for asymptomatic carriage in ongoing transmission for both outbreak and non-outbreak settings [5]. This could be facilitated by environmental contamination or other indirect transmission pathways. Such transmission pathways, as well as person-to-person routes, have been identified as potential transmission mechanisms of *C. difficile* in paediatric populations [64,65]. Healthcare premises have been potentially further implicated in CDI, with one study on Serbian children with community-onset CDI showing that 73 % of patients had exposure to healthcare facilities <12 weeks prior to CDI onset. The authors acknowledge however that the exposure to healthcare facilities may simply reflect the presence of underlying conditions which confer a higher risk of CDI [66]. Transmission of *C. difficile* amongst paediatric CDI patients was investigated in one study, with 131 isolates from 107 patients over a 12 month period identified. Of these isolates, 104 were genetically distinct, with only eight isolates found to cause CDI in more than one patient. The authors identified direct/indirect transmission events as only occurring in 12 % of cases, lower than the frequency of transmission occurring in adult CDI populations [67]. Onward transmission from infants to adult patients has been documented, with two cases of mothers developing CDI with the same *C. difficile* immunoblot types carried asymptomatically by their infants [68].

Outbreaks of CDI in paediatric populations are rarely described, despite relatively high *C. difficile* carriage rates, although the sensitivity of this dataset may be influenced by diagnostic testing algorithms [69]. One study identified three CDI cases diagnosed within 48 h on a paediatric haematology/oncology unit, although all three patient isolates had distinct PFGE patterns [70]. Similarly, one article reported three cases of CDI diagnosed within 15 days in a paediatric intensive care setting, with two different ribotypes detected. The authors here note that one of the isolates was the same ribotype often seen in their adult populations, indicating transmission across patient populations [71]. A retrospective analysis of CDI patients in a paediatric oncology centre identified 26 isolates from 27 patients over a 3 year, 10-month period. High genetic diversity of isolates was identified, with ribotype 027 being absent, despite authors commenting that this is the dominant ribotype in their adult populations [72]. Another retrospective analysis on frozen stool samples showed that a suspected transmission chain of * C. difficile* consisted of genetically distinct isolates. The authors comment however on two clusters of closely related isolates (0–4 and 4–7 loci variation when typed by double locus sequence typing) which, when combined with epidemiological evidence, was suggestive of transmission events [73]. A further retrospective cohort study over a 4-year timeframe with 299 patients analysed determined that 24 % of all *C. difficile* isolates belonged to the ribotype 014 (NAP4), which had a relapse rate of 41 % – compared to 15 % in other strains [74]. This strain has been further associated with paediatric CDI, with one retrospective study reporting two patients as having the NAP4 strain – with one patient being shown to shed this strain into the hospital environment 51 days post-CDI diagnosis [75]. Occasionally small-scale CDI outbreaks with a single clone as the causative agent are reported in paediatric populations. One article documents seven healthcare-onset CDI cases, with all isolates displaying the same PCR riobtype [76]. A further study identified six CDI patients in a paediatric orthopaedic service, with 11 collected isolates all belonging to serogroup C [77].

## Indirect transmission risks

Contamination of the clinical environment with *C. difficile* is well understood to occur when patients are positive for *C. difficile*, either by infection or colonization. *C. difficile* spores are able to persist in the clinical environment for extended periods of time and resist many forms of decontamination. Environmental contamination in healthcare settings is thought to contribute to the colonization of the neonatal gut with *C. difficile* and therefore environmental loading may present an ongoing transmission risk within healthcare environments [8]. Historic evidence has illustrated that the majority of environmental isolates recovered from surfaces, medical equipment and hands in a neonatal unit belonged to the same serogroups as neonatal faecal isolates [78]. Recent investigations into environmental contamination patterns of *C. difficile* in paediatric clinical settings have shown relatively low *C. difficile* environmental burdens. One study identified five surfaces as positive for *C. difficile* in a haematology/oncology unit, with sampling performed in response to three cases of CDI being diagnosed in 48 h. The environmental isolates had different PFGE patterns to the patient isolates, with 4/5 environmental isolates sharing the same PFGE pattern [70]. One investigation recovered *C. difficile* from 7/100 samples from the ward environments and staff clothing on a gastroenterology and an oncology unit. Five of the seven isolates here were determined to be toxigenic, with one environmental isolate being ribotype 027 [79]. Further research identified high rates of asymptomatic carriage of *C. difficile* in a neonatal intensive care unit (NICU) (25.7%), with 2 out of 29 associated environmental sites detected positive for *C. difficile*. Follow-on environmental sampling identified 5/10 diaper scales were contaminated with *C. difficile*, with the study highlighting the potential for neonates in an NICU setting to act as asymptomatic reservoirs for *C. difficile* shedding into the environment [80].

Due to the persistence of *C. difficile* spores in the environment, infection control interventions which target environmental contamination are a key strategy to prevent CDI. A study modelling the transmission of *C. difficile* in a paediatric unit *in sillico* determined that daily environmental decontamination with a sporicidal agent was the most effective way to reduce both healthcare-onset CDI and *C. difficile* colonization [64]. The authors identified that person-to-environment transfer efficiency of *C. difficile* was one of the most influential parameters impacting the output of the model, alongside person-to-person transfer efficiency. As the study relates to a computer model, the findings are limited by the parameters set for the model which the authors acknowledge are derived from investigations in *C. difficile* transmission in adult hospitals [64]. Due to differences in how paediatric patients interact with their environment, environmental contamination may play a more significant role in paediatric settings.

Controlling the presence of *C. difficile* spores in the clinical environment is critical in the prevention of *C. difficile* transmission. This is achieved with the use of sporicidal cleaning agents, with chlorine-based cleaning agents well known to effectively eliminate *C. difficile* spores and reduce CDI rates [81]. Such cleaning measures are recommended for *C. difficile* transmission mitigation in paediatric settings [19]. In order to further support environmental cleaning practices, decontamination utilizing UV-C emitting devices can be used. Conflicting evidence on their efficacy at reducing CDI rates exists however, with studies concluding that their use reduces healthcare associated CDI [82], while others showing no change in CDI rates following UV-C usage cessation [83]. Furthermore, a cluster randomized crossover-controlled trial on four cancer and one solid organ transplant units identified no significant reduction in CDI rates when UV-C decontamination was used as a component of daily and terminal cleans [84]. The authors here do note that the lack of significant reduction may be due to the existing low rates of CDI in the study setting. Few studies have investigated the impact of UV-C usage in decontamination in paediatric settings, with further research required to fully elucidate this. Similarly, hydrogen peroxide technologies have been shown to reduce *C. difficile* infection rates within paediatric settings. One study illustrated a statistically significant reduction in *Clostridioides*-associated gastroenteritis when dry hydrogen peroxide was used as a cleaning adjunct compared to the control unit [85]. Hydrogen peroxide is well understood to be effective at reducing environmental loading of *C. difficile* spores, however additional research into their application within paediatric settings is required [81].

## Conclusions

*C. difficile* is an organism of increasing interest within paediatric settings, both within community and acute healthcare. Despite this, elucidating the extent of its impact is challenging, due to a number of factors. These include variability in classification of CDI, poor understanding of the role of colonization in both progression to disease and role in transmission risk, and variability in diagnostic approaches and workflows.

Although approaches to managing *C. difficile* within the paediatric population have frequently been extrapolated from data based within adult settings. However, age dependent colonization, differences in the way that children interact with the environment, and reduction in the comparative number of monoclonal outbreaks suggest that *C. difficile* infection and detection should be further investigated and potentially managed differently to approaches seen within adult healthcare.

A lack of dedicated paediatric studies mean that clinical management information related to the best way to treat individual patients or understand the role of colonization is inconsistent. This knowledge gap needs to be addressed in order to develop paediatric specific guidance to facilitate improvements in practice.

## Abbreviations

AAD: antibiotic associated diarrhoea
CDI: *Clostridioides difficile* infection
EIA: enzyme immunoassays
FMT: faecal microbial transplantation
GDH: glutamate dehydrogenase
IDSA: Infectious Diseases Society of America
MLST: multi-locus sequence typing
NICE: National Institute of Health and Care Excellence
NICU: neonatal intensive care unit
PCR: polymerase chain reaction
PFGE: pulse field gel electrophoresis
rCDI: recurrent *Clostridioides difficile* infection
REA: restriction endonuclease analysis
SHEA: Society for Healthcare Epidemiology of America
WGS: whole genome sequencing

## References

[1] Rodriguez C, Van Broeck J, Taminiau B, Delmée M, Daube G (2016). *Clostridium difficile* infection: early history, diagnosis and molecular strain typing methods. Microb Pathog.

[2] Perumalsamy S, Riley TV (2021). Molecular epidemiology of *Clostridioides difficile* infections in children. J Pediatric Infect Dis Soc.

[3] Fatima R, Aziz M (2019). The hypervirulent strain of *Clostridium difficile*: NAP1/B1/027 - a brief overview. Cureus.

[4] Shaw HA, Preston MD, Vendrik KEW, Cairns MD, Browne HP (2020). The recent emergence of a highly related virulent *Clostridium difficile* clade with unique characteristics. Clin Microbiol Infect.

[5] Crobach MJT, Vernon JJ, Loo VG, Kong LY, Péchiné S (2018). Understanding *Clostridium difficile* colonization. Clin Microbiol Rev.

[6] al Saif N, Brazier JS (1996). The distribution of *Clostridium difficile* in the environment of South Wales. J Med Microbiol.

[7] Weese JS, Finley R, Reid-Smith RR, Janecko N, Rousseau J (2010). Evaluation of *Clostridium difficile* in dogs and the household environment. Epidemiol Infect.

[8] Borali E, Giacomo C, De (2016). *Clostridium difficile* infection in children: a review. J Pediatr Gastroenterol Nutr.

[9] Abad-Fau A, Sevilla E, Martín-Burriel I, Moreno B, Bolea R (2023). Update on commonly used molecular typing methods for *Clostridioides difficile*. Microorganisms.

[10] Curry SR, Muto CA, Schlackman JL, Pasculle AW, Shutt KA (2013). Use of multilocus variable number of tandem repeats analysis genotyping to determine the role of asymptomatic carriers in *Clostridium difficile* transmission. Clin Infect Dis.

[11] Al-Jumaili IJ, Shibley M, Lishman AH, Record CO (1984). Incidence and origin of *Clostridium difficile* in neonates. J Clin Microbiol.

[12] Jangi S, Lamont JT (2010). Asymptomatic colonization by *Clostridium difficile* in infants: implications for disease in later life. J Pediatr Gastroenterol Nutr.

[13] Stoesser N, Eyre DW, Quan TP, Godwin H, Pill G (2017). Epidemiology of *Clostridium difficile* in infants in Oxfordshire, UK: risk factors for colonization and carriage, and genetic overlap with regional *C. difficile* infection strains. PLoS One.

[14] Enoch DA, Butler MJ, Pai S, Aliyu SH, Karas JA (2011). *Clostridium difficile* in children: colonisation and disease. J Infect.

[15] Tilkorn FKMT, Frickmann H, Simon IS, Schwanbeck J, Horn S (2020). Antimicrobial resistance patterns in *Clostridioides difficile* strains isolated from neonates in Germany. Antibiotics.

[16] Wendt JM, Cohen JA, Mu Y, Dumyati GK, Dunn JR (2014). *Clostridium difficile* infection among children across diverse US geographic locations. Pediatrics.

[17] Krutova M, de Meij TGJ, Fitzpatrick F, Drew RJ, Wilcox MH (2022). How to: *Clostridioides difficile* infection in children. Clin Microbiol Infect.

[18] Lessa FC, Mu Y, Bamberg WM, Beldavs ZG, Dumyati GK (2015). Burden of *Clostridium difficile* infection in the United States. N Engl J Med.

[19] Sammons JS, Toltzis P, Zaoutis TE (2013). *Clostridium difficile* infection in children. JAMA Pediatr.

[20] Davies KA, Ashwin H, Longshaw CM, Burns DA, Davis GL (2016). Diversity of Clostridium difficile PCR ribotypes in Europe: results from the European, multicentre, prospective, biannual, point-prevalence study of *Clostridium difficile* infection in hospitalised patients with diarrhoea (EUCLID), 2012 and 2013. Eurosurveillance.

[21] Sandora TJ, Fung M, Flaherty K, Helsing L, Scanlon P (2011). Epidemiology and risk factors for *Clostridium difficile* infection in children. Pediatr Infect Dis J.

[22] Morinville V, McDonald J (2005). *Clostridium difficile*-associated diarrhea in 200 Canadian children. Can J Gastroenterol.

[23] Balassiano IT, Yates EA, Domingues R, Ferreira EO (2012). *Clostridium difficile*: a problem of concern in developed countries and still a mystery in Latin America. J Med Microbiol.

[24] Smits WK, Lyras D, Lacy DB, Wilcox MH, Kuijper EJ (2016). *Clostridium difficile* infection. Nat Rev Dis Primers.

[25] Lawler AJ, Lambert PA, Worthington T (2020). A revised understanding of *Clostridioides difficile* spore germination. Trends Microbiol.

[26] Ihekweazu FD, Versalovic J (2018). Development of the pediatric Gut microbiome: impact on health and disease. Am J Med Sci.

[27] Romyasamit C, Thatrimontrichai A, Aroonkesorn A, Chanket W, Ingviya N (2020). *Enterococcus faecalis* isolated from infant feces inhibits toxigenic *Clostridioides (Clostridium) difficile*. Front Pediatr.

[28] Burnham C-AD, Carroll KC (2013). Diagnosis of *Clostridium difficile* infection: an ongoing conundrum for clinicians and for clinical laboratories. Clin Microbiol Rev.

[29] Shin JH, Chaves-Olarte E, Warren CA (2016). *Clostridium difficile* infection. Microbiol Spectr.

[30] Eckert C, Emirian A, Le Monnier A, Cathala L, De Montclos H (2015). Prevalence and pathogenicity of binary toxin-positive *Clostridium difficile* strains that do not produce toxins A and B. New Microbes New Infect.

[31] Zhang Y, Li S, Yang Z, Shi L, Yu H (2018). Cysteine protease-mediated autocleavage of *Clostridium difficile* toxins regulates their proinflammatory activity. Cell Mol Gastroenterol Hepatol.

[32] Bobo LD, El Feghaly RE, Chen Y-S, Dubberke ER, Han Z (2013). MAPK-activated protein kinase 2 contributes to *Clostridium difficile*-associated inflammation. Infect Immun.

[33] Kuiper G-A, van Prehn J, Ang W, Kneepkens F, van der Schoor S (2017). *Clostridium difficile* infections in young infants: case presentations and literature review. IDCases.

[34] Manzoor F, Manzoor S, Pinto R, Brown K, Langford BJ (2023). Does this patient have *Clostridioides difficile* infection? A systematic review and meta-analysis. Clin Microbiol Infect.

[35] Kohler CM, Quintanar Alfaro AG, Hayden RT, Margolis EB (2022). Real-time quantitative PCR method for detection and quantification of *Clostridioides difficile* cells and spores. J Microbiol Methods.

[36] McDonald LC, Gerding DN, Johnson S, Bakken JS, Carroll KC (2018). Clinical practice guidelines for *Clostridium difficile* infection in adults and children: 2017 update by the Infectious Diseases Society of America (IDSA) and Society for Healthcare Epidemiology of America (SHEA). Clin Infect Dis.

[37] Department of Health (2012). Updated guidance on the diagnosis and reporting of *Clostridium difficile*. https://www.gov.uk/government/publications/updated-guidance-on-the-diagnosis-and-reporting-of-clostridium-difficile.

[38] Carroll KC (2011). Tests for the diagnosis of *Clostridium difficile* infection: the next generation. Anaerobe.

[39] Luna RA, Boyanton BL, Mehta S, Courtney EM, Webb CR (2011). Rapid stool-based diagnosis of *Clostridium difficile* infection by real-time PCR in a children’s hospital. J Clin Microbiol.

[40] Kociolek LK, Crews JD, Schwenk HT (2021). Recent advances in *Clostridioides difficile* infection epidemiology, diagnosis and treatment in children. Curr Opin Infect Dis.

[41] Polage CR, Gyorke CE, Kennedy MA, Leslie JL, Chin DL (2015). Overdiagnosis of *Clostridium difficile* infection in the molecular test era. JAMA Intern Med.

[42] Qutub M, Govindan P, Vattappillil A (2019). Effectiveness of a two-step testing algorithm for reliable and cost-effective detection of *Clostridium difficile* infection in a tertiary care hospital in Saudi Arabia. Med Sci.

[43] Guarino A, Ashkenazi S, Gendrel D, Lo Vecchio A, Shamir R (2014). European Society for pediatric gastroenterology, hepatology, and Nutrition/European Society for Pediatric Infectious Diseases evidence-based guidelines for the management of acute gastroenteritis in children in Europe: update 2014. J Pediatr Gastroenterol Nutr.

[44] Pohl JF (2012). Clostridium difficile infection and proton pump inhibitors. Curr Opin Pediatr.

[45] National Institute for Health and Care Excellence (2021). Clostridioides difficile infection: antimicrobial prescribing, NG199. https://www.nice.org.uk/guidance/ng199.

[46] Johnson S, Lavergne V, Skinner AM, Gonzales-Luna AJ, Garey KW (2021). Clinical Practice Guideline by the Infectious Diseases Society of America (IDSA) and Society for Healthcare Epidemiology of America (SHEA): 2021 focused update guidelines on management of *Clostridioides difficile* infection in adults. Clin Infect Dis.

[47] Sammons JS, Gerber JS, Tamma PD, Sandora TJ, Beekmann SE (2014). Diagnosis and management of *Clostridium difficile* infection by pediatric infectious diseases physicians. J Pediatric Infect Dis Soc.

[48] Cohen SH, Gerding DN, Johnson S, Kelly CP, Loo VG (2010). Clinical practice guidelines for *Clostridium difficile* infection in adults: 2010 update by the society for healthcare epidemiology of America (SHEA) and the infectious diseases society of America (IDSA). Infect Control Hosp Epidemiol.

[49] Yin J, Kociolek LK, Same RG, Hsu AJ, Amoah J (2019). Oral vancomycin may be associated with earlier symptom resolution than metronidazole for hospitalized children with nonsevere *Clostridiodes difficile* infections. Open Forum Infect Dis.

[50] Mezoff E, Mann EA, Hart KW, Lindsell CJ, Cohen MB (2011). *Clostridium difficile* infection and treatment in the pediatric inflammatory bowel disease population. J Pediatr Gastroenterol Nutr.

[51] British National Formulary for Children (2023). Fidaxomicin. https://bnfc.nice.org.uk/drugs/fidaxomicin/.

[52] Skinner AM, Scardina T, Kociolek LK (2020). Fidaxomicin for the treatment of *Clostridioides difficile* in children. Future Microbiol.

[53] Oliver MB, Vaughn BP (2022). Fidaxomicin use in the pediatric population with *Clostridioides difficile*. Clin Pharmacol.

[54] Wolf J, Kalocsai K, Fortuny C, Lazar S, Bosis S (2020). Safety and efficacy of fidaxomicin and vancomycin in children and adolescents with *Clostridioides* (*Clostridium*) *difficile* infection: a phase 3, multicenter, randomized, single-blind clinical trial (SUNSHINE). Clin Infect Dis.

[55] Gawronska A, Banasiuk M, Lachowicz D, Pituch H, Albrecht P (2017). Metronidazole or rifaximin for treatment of *Clostridium difficile* in pediatric patients with inflammatory bowel disease: a randomized clinical trial. Inflamm Bowel Dis.

[56] Sferra TJ, Merta T, Neely M, Murta de Oliveira C, Lassaletta A (2023). Double-blind, placebo-controlled study of bezlotoxumab in children receiving antibacterial treatment for *Clostridioides difficile* infection (MODIFY III). J Pediatric Infect Dis Soc.

[57] Faust SN, Wilcox MH, Banaszkiewicz A, Bouza E, Raymond J (2015). Lack of evidence for an unmet need to treat *Clostridium difficile* infection in infants aged <2 years: expert recommendations on how to address this issue. Clin Infect Dis.

[58] Fareed S, Sarode N, Stewart FJ, Malik A, Laghaie E (2018). Applying fecal microbiota transplantation (FMT) to treat recurrent *Clostridium difficile* infections (rCDI) in children. PeerJ.

[59] Tun KM, Hsu M, Batra K, Lo C-H, Laeeq T (2022). Efficacy and safety of fecal microbiota transplantation in treatment of *Clostridioides difficile* infection among pediatric patients: a systematic review and meta-analysis. Microorganisms.

[60] Szajewska H, Canani RB, Guarino A, Hojsak I, Indrio F (2016). Probiotics for the prevention of antibiotic-associated diarrhea in children. J Pediatr Gastroenterol Nutr.

[61] Goldenberg JZ, Yap C, Lytvyn L, Lo CK-F, Beardsley J (2017). Probiotics for the prevention of *Clostridium difficile*-associated diarrhea in adults and children. Cochrane Database Syst Rev.

[62] Atıcı S, Soysal A, Karadeniz Cerit K, Yılmaz Ş, Aksu B (2017). Catheter-related *Saccharomyces cerevisiae* fungemia following *Saccharomyces boulardii* probiotic treatment: in a child in intensive care unit and review of the literature. Med Mycol Case Rep.

[63] Blixt T, Gradel KO, Homann C, Seidelin JB, Schønning K (2017). Asymptomatic carriers contribute to nosocomial *Clostridium difficile* infection: a cohort study of 4508 patients. Gastroenterology.

[64] Barker AK, Scaria E, Alagoz O, Sethi AK, Safdar N (2020). Reducing *C. difficile* in children: an agent-based modeling approach to evaluate intervention effectiveness. Infect Control Hosp Epidemiol.

[65] Matsuki S, Ozaki E, Shozu M, Inoue M, Shimizu S (2005). Colonization by *Clostridium difficile* of neonates in a hospital, and infants and children in three day-care facilities of Kanazawa, Japan. Int Microbiol.

[66] Predrag S, Branislava K, Nikola S, Niko R, Zorica S-R (2018). Community-acquired *Clostridium difficile* infection in Serbian pediatric population. Eur J Clin Microbiol Infect Dis.

[67] Kociolek LK, Gerding DN, Espinosa RO, Patel SJ, Shulman ST (2018). *Clostridium difficile* whole genome sequencing reveals limited transmission among symptomatic children: a single-center analysis. Clin Infect Dis.

[68] McFarland LV, Surawicz CM, Greenberg RN, Bowen KE, Melcher SA (1999). Possible role of cross-transmission between neonates and mothers with recurrent *Clostridium difficile* infections. Am J Infect Control.

[69] McFarland LV, Ozen M, Dinleyici EC, Goh S (2016). Comparison of pediatric and adult antibiotic-associated diarrhea and *Clostridium difficile* infections. World J Gastroenterol.

[70] Warrack S, Duster M, Van Hoof S, Schmitz M, Safdar N (2014). *Clostridium difficile* in a children’s hospital: assessment of environmental contamination. Am J Infect Control.

[71] Bustinza A, Solana MJ, Padilla B, López-Herce J, Santiago MJ (2009). Nosocomial outbreak of *Clostridium difficile*-associated disease in a pediatric intensive care unit in Madrid. Infect Control Hosp Epidemiol.

[72] Simon A, Mock M, Graf N, von Müller L (2018). Investigation of *Clostridium difficile* ribotypes in symptomatic patients of a German pediatric oncology center. Eur J Pediatr.

[73] Blanc DS, Poncet F, Grandbastien B, Prod’hom G, Greub G (2022). Molecular typing of *Clostridioides difficile* from frozen stool samples to investigate cross-transmissions: a proof of concept. Indian J Med Microbiol.

[74] Schwartz KL, Darwish I, Richardson SE, Mulvey MR, Thampi N (2014). Severe clinical outcome is uncommon in *Clostridium difficile* infection in children: a retrospective cohort study. BMC Pediatr.

[75] Dantes R, Epson EE, Dominguez SR, Dolan S, Wang F (2016). Investigation of a cluster of *Clostridium difficile* infections in a pediatric oncology setting. Am J Infect Control.

[76] Cartwright CP, Stock F, Beekmann SE, Williams EC, Gill VJ (1995). PCR amplification of rRNA intergenic spacer regions as a method for epidemiologic typing of *Clostridium difficile*. J Clin Microbiol.

[77] Ferroni A, Merckx J, Ancelle T, Pron B, Abachin E (1997). Nosocomial outbreak of *Clostridium difficile* diarrhea in a pediatric service. Eur J Clin Microbiol Infect Dis.

[78] Delmée M, Verellen G, Avesani V, Francois G (1988). *Clostridium difficile* in neonates: serogrouping and epidemiology. Eur J Pediatr.

[79] Lemiech-Mirowska E, Michałkiewicz M, Sierocka A, Gaszyńska E, Marczak M (2023). The hospital environment as a potential source for *Clostridioides difficile* transmission based on spore detection surveys conducted at paediatric oncology and gastroenterology units. Int J Environ Res Public Health.

[80] Faden HS, Dryja D (2015). Importance of asymptomatic shedding of *Clostridium difficile* in environmental contamination of a neonatal intensive care unit. Am J Infect Control.

[81] Barbut F (2015). How to eradicate *Clostridium difficile* from the environment. J Hosp Infect.

[82] Steele M, Hurtado RR, Rychlik K, Bonebrake A, Bovee MC (2021). Impact of an automated multiple emitter whole-room ultraviolet-C disinfection system on hospital acquired infections: a quasi-experimental study. Am J Infect Control.

[83] Abosi OJ, Kobayashi T, Holley S, Kukla ME, Dains A (2021). Stable *Clostridioides difficile* infection rates after the discontinuation of ultraviolet light for terminal disinfection at a tertiary care center, Iowa 2019-2020. Am J Infect Control.

[84] Rock C, Hsu YJ, Curless MS, Carroll KC, Ross Howard T (2022). Ultraviolet-C light evaluation as adjunct disinfection to remove multidrug-resistant organisms. Clin Infect Dis.

[85] Melgar M, Ramirez M, Chang A, Antillon F (2022). Impact of dry hydrogen peroxide on hospital-acquired infection at a pediatric oncology hospital. Am J Infect Control.

[86] El-Matary W, Nugent Z, Yu BN, Lix LM, Targownik LE (2019). Trends and predictors of *Clostridium difficile* infection among children: a Canadian population-based study. J Pediatr.

[87] Jimenez J, Drees M, Loveridge-Lenza B, Eppes S, delRosario F (2015). Exposure to gastric acid-suppression therapy is associated with health care- and community-associated *Clostridium difficile* Infection in children. J Pediatr Gastroenterol Nutr.

[88] Tariq R, Singh S, Gupta A, Pardi DS, Khanna S (2017). Association of gastric acid suppression with recurrent *Clostridium difficile* infection: a systematic review and meta-analysis. JAMA Intern Med.

[89] Ochfeld E, Balmert LC, Patel SJ, Muller WJ, Kociolek LK (2019). Risk factors for Clostridioides (*Clostridium*) *difficile* infection following solid organ transplantation in children. Transpl Infect Dis.

[90] Parmar D, Dang R, Miranda-Katz M, Alabaster A, Greenhow TL (2019). Risk factors for recurrent community-associated *Clostridiodes difficile* infection in children. Pediatr Infect Dis J.

[91] Balram B, Khoury AA, Lakatos PL, Bessissow T (2018). 276 - risk factors and outcomes associated with *Clostridium difficile* infection in inflammatory bowel disease: a systematic review and meta-analysis. Gastroenterology.

[92] Kociolek LK, Palac HL, Patel SJ, Shulman ST, Gerding DN (2015). Risk factors for recurrent *Clostridium difficile* infection in children: a nested case-control study. J Pediatr.

[93] Armin S, Shamsian S, Drakhshanfar H (2013). Colonization with *Clostridium difficile* in children with cancer. Iran J Pediatr.

[94] Freeman J, Bauer MP, Baines SD, Corver J, Fawley WN (2010). The changing epidemiology of *Clostridium difficile* infections. Clin Microbiol Rev.

[95] Ramírez-Vargas G, López-Ureña D, Badilla A, Orozco-Aguilar J, Murillo T (2018). Novel Clade C-I *Clostridium difficile* strains escape diagnostic tests, differ in pathogenicity potential and carry toxins on extrachromosomal elements. Sci Rep.

